# Neuro-Ophthalmological Disorders Associated with Obstructive Sleep Apnoea

**DOI:** 10.3390/ijms26146649

**Published:** 2025-07-11

**Authors:** Snježana Kaštelan, Lea Kozina, Maja Alaber, Zora Tomić, Marina Andrešić, Ivana Bakija, Diana Bućan, Tomislav Matejić, Domagoj Vidović

**Affiliations:** 1 Department of Ophthalmology, Clinical Hospital Dubrava, School of Medicine, University of Zagreb, 10000 Zagreb, Croatia; 2 Department of Sleep Disorders, University Psychiatric Hospital Vrapče, 10000 Zagreb, Croatia; 3 Department of Emergency Medicine of Bjelovar-Bilogora County, 43000 Bjelovar, Croatia; 4 Health Centre of the Croatian Department of Internal Affairs, 10000 Zagreb, Croatia; 5 Department of Endocrinology, Diabetes and Metabolic Disease, Clinical Hospital “Sestre Milosrdnice”, 10000 Zagreb, Croatia; 6 Department of Integrative Psychiatry, Psychiatry Hospital “Sveti Ivan”, 10090 Zagreb, Croatia; 7 Occupational Medicine Practices, 21000 Split, Croatia; 8 Surgery Clinic, Clinical Hospital Sveti Duh, 10000 Zagreb, Croatia

**Keywords:** obstructive sleep apnoea, neuro-ophthalmology, molecular mechanisms, intermittent hypoxia, oxidative stress, intracranial pressure, optic nerve, glaucoma, visual function, retinal changes

## Abstract

Obstructive sleep apnoea (OSA) is a prevalent condition characterised by intermittent upper airway obstruction during sleep, resulting in recurrent hypoxia and sleep fragmentation. Emerging evidence highlights the significant impact of OSA on neuro-ophthalmological health, linking it to conditions such as glaucoma, optic neuropathy, papilledema, and visual field defects. These associations emphasise the importance of understanding the mechanisms connecting OSA to neuro-ophthalmological disorders to enhance early diagnosis and management. This review explores the pathophysiological pathways, including hypoxia-induced vascular dysregulation, oxidative stress, inflammation, and intracranial pressure fluctuations, that contribute to ocular and neurological impairments in OSA patients. Advanced diagnostic tools, such as optical coherence tomography and polysomnography, offer promising avenues for detecting subclinical neuro-ophthalmological changes, enabling timely intervention. Management strategies, primarily centred on continuous positive airway pressure therapy, have shown efficacy in mitigating OSA-related neuro-ophthalmological complications. However, surgical and pharmacological interventions and lifestyle modifications remain vital components of a multidisciplinary approach to care. Despite advancements, significant research gaps persist, particularly in understanding the long-term impact of OSA treatment on neuro-ophthalmological outcomes and identifying specific biomarkers for early detection. Future research should prioritise longitudinal studies, interdisciplinary collaborations, and personalised medicine approaches to address these challenges. Recognising and treating neuro-ophthalmological disorders in OSA patients is imperative for improving quality of life and preventing irreversible visual and neurological damage.

## 1. Introduction

Obstructive sleep apnoea (OSA) is a common sleep-related breathing disorder characterised by repeated episodes of upper airway collapse during sleep. Complete obstruction (apnoea) is a transient cessation of spontaneous respiration lasting at least 10 s. In contrast, partial obstruction (hypopnoea) involves a ≥30% reduction in airflow for the same duration, accompanied by oxygen desaturation or arousal from sleep [1]. These recurrent respiratory disturbances lead to intermittent hypoxia, sleep fragmentation, frequent nocturnal arousals, and sustained sympathetic overactivity, contributing to systemic and organ-specific dysfunction [2]. Despite its high prevalence and multisystem impact, OSA often goes undiagnosed and undertreated, particularly in individuals presenting with non-respiratory or atypical symptoms.

### 1.1. Diagnostic Protocol

Polysomnography remains the gold standard for diagnosing OSA and quantifying its severity through the apnoea–hypopnoea index (AHI), which indicates the average number of apnoeic and hypopnoeic events per hour of sleep [2,3]. Current clinical guidelines classify OSA severity based on the following AHI thresholds: 5–15 events/hour indicates mild OSA, 15–30 moderate, and over 30 severe disease (Table 1) [4]. Screening tools such as the Epworth Sleepiness Scale, STOP-Bang, and the Berlin Questionnaire are useful instruments for risk stratification in primary care and specialist settings; however, they lack the sensitivity and specificity of objective measures such as polysomnography [5].

The diagnostic workup for OSA begins with clinical suspicion based on hallmark symptoms such as loud snoring, witnessed apnoeas, nocturnal choking, and excessive daytime sleepiness. Initial risk stratification is commonly conducted using validated questionnaires, including the Epworth Sleepiness Scale, STOP-Bang, and the Berlin Questionnaire. While these tools are helpful in screening, definitive diagnosis requires objective testing. Polysomnography is the gold-standard diagnostic modality that involves the overnight monitoring of multiple physiological parameters, including airflow, oxygen saturation, respiratory effort, heart rate, electroencephalography, and limb movements. The AHI, derived from polysomnography, quantifies OSA severity [1,2,3,4]. Home sleep apnoea testing (HSAT) may be appropriate for patients with a high pretest probability and no significant comorbidities [6].

The diagnostic algorithm also includes evaluating anatomical contributors via upper airway endoscopy or imaging, especially when surgical interventions are considered. In select patients, overnight oximetry, capnography, and oesophageal pressure monitoring may provide further insights. Phenotypic and endotypic assessments, including loop gain, arousal threshold, and airway collapsibility, increasingly guide personalised therapeutic strategies [4].

Advancements in ocular imaging and functional diagnostics have improved the early detection of subclinical neuro-ophthalmological changes in patients with OSA. Optical coherence tomography (OCT) and OCT angiography (OCTA) enable a non-invasive assessment of retinal nerve fibre layer (RNFL) thinning, optic disc morphology, and microvascular rarefaction before the appearance of clinical symptoms or visual loss. Functional assessments such as visual field testing and electrophysiological evaluations further delineate visual pathway dysfunction. When integrated with polysomnographic metrics, these tools help identify individuals at highest risk for vision-threatening complications [7].

### 1.2. Epidemiology

Epidemiological data estimates that OSA affects approximately 9–38% of the adult population, with a significantly higher prevalence in males (13–33%) compared to females (6–19%) [8]. Risk factors include male sex, increasing age, obesity, craniofacial anomalies, and adenotonsillar hypertrophy [9,10]. In addition to its negative effects on sleep quality, OSA is closely associated with numerous systemic conditions, including cardiovascular diseases (hypertension, myocardial infarction, stroke, atrial fibrillation), metabolic disorders such as type 2 diabetes, and cognitive impairment [11].

### 1.3. Impact on the Visual System

Although the systemic complications of OSA are well established, its impact on the visual system, particularly the neuro-ophthalmological structures, remains underappreciated. Emerging evidence links OSA to a spectrum of neuro-ophthalmological disorders, including non-arteritic anterior ischaemic optic neuropathy (NAION), glaucoma, particularly normal-tension glaucoma (NTG), papilloedema secondary to raised intracranial pressure, diabetic retinopathy, central serous chorioretinopathy, and idiopathic intracranial hypertension (IIH) [12,13]. These associations highlight the importance of elucidating the pathophysiological mechanisms by which sleep-disordered breathing impacts ocular and systemic health.

### 1.4. Mechanisms Linking OSA to Neuro-Ophthalmological Disorders

The mechanisms linking OSA to neuro-ophthalmological disorders are complex and multifactorial, involving intermittent hypoxia, oxidative stress, intracranial pressure fluctuations, systemic inflammation, and vascular dysregulation [14]. Intermittent hypoxia during apnoeic episodes activates hypoxia-inducible factor 1α (HIF-1α), a key transcription factor that regulates the expression of over 100 genes related to cellular adaptation to low oxygen [15]. One of the major HIF-1α target genes is vascular endothelial growth factor (VEGF), a potent angiogenic mediator implicated in the development of proliferative diabetic retinopathy [16,17]. Simultaneously, mitochondrial dysfunction and immune cell activation during hypoxic stress led to the generation of reactive oxygen species (ROS), contributing to retinal ganglion cell apoptosis and trabecular meshwork damage, key features of glaucomatous optic neuropathy and elevated intraocular pressure [18,19,20]. Furthermore, recurrent apnoeic episodes cause sharp fluctuations in intrathoracic pressure, resulting in hypercapnia, cerebral vasodilation, and altered intracranial pressure dynamics. These hemodynamic changes may impair optic nerve perfusion and promote papilloedema in susceptible individuals [12].

OSA-related neurodegeneration also elicits a systemic inflammatory response, characterised by elevated levels of proinflammatory cytokines, including interleukin-6 (IL-6), tumour necrosis factor-α (TNF-α), IL-8, and C-reactive protein (CRP) [15,21]. This inflammatory environment increases the expression of vascular adhesion molecules such as intercellular adhesion molecule 1 (ICAM-1) and vascular cell adhesion protein 1 (VCAM-1) on endothelial surfaces [22]. Their upregulation impairs endothelial function, diminishes vasodilator availability, and promotes vasoconstriction, mechanisms that may contribute to NAION, diabetic microangiopathy, and glaucomatous damage [12].

### 1.5. Aims of This Review

Despite growing recognition of the relationship between OSA and neuro-ophthalmological disorders, substantial gaps remain in our understanding. Prospective longitudinal studies evaluating the effects of OSA treatment on visual outcomes are limited. Additionally, heterogeneity in patient populations, diagnostic definitions, and outcome measures have impeded the development of unified clinical management guidelines [7,12,13,14,23]. Addressing these gaps will require interdisciplinary collaboration among sleep medicine specialists, ophthalmologists, neurologists, and basic researchers.

In this context, this review aims to synthesise the current evidence on the neuro-ophthalmological manifestations of OSA. It will explore its underlying pathophysiological pathways, assess the utility of diagnostic imaging and functional testing, and evaluate the current state of therapeutic strategies. A deeper understanding of these interrelated mechanisms will pave the way for earlier detection, targeted intervention, and improved outcomes in patients with coexisting OSA and neuro-ophthalmological disease.

### 1.6. Data Collection

A systematic review was carried out through an extensive literature search of the MEDLINE and PubMed databases, encompassing publications up to May 2025. The search strategy used a combination of the following keywords: “obstructive sleep apnoea”, “neuro-ophthalmological disorders”, “pathophysiology”, “optic neuropathy”, “NAION”, “glaucoma”, “papilloedema”, “intracranial hypertension”, “optic nerve”, “visual function”, “retinal changes”, “diabetic retinopathy”, “oculomotor dysfunction”, “CPAP”, and “polysomnography”. The search was restricted to studies published in English. After removing duplicates, full-text articles were retrieved and assessed for relevance based on their titles and abstracts. Reference lists of the selected articles were also manually screened to identify additional relevant studies not captured in the initial search. Studies meeting the inclusion criteria were qualitatively evaluated, focusing on those published within the last 15 years to ensure the most recent advances were included. No quantitative meta-analysis was performed, as the aim was to provide a thorough narrative synthesis of the current evidence.

## 2. Pathophysiology of Obstructive Sleep Apnoea

OSA is characterised by recurrent episodes of partial or complete upper airway obstruction during sleep, resulting in intermittent hypoxia (IH), hypercapnia, oxygen desaturation, and frequent arousals. These events disrupt normal sleep architecture, leading to fragmentation and widespread systemic consequences [15,24].

### 2.1. Structural and Functional Mechanisms of Upper Airway Collapse

The pathogenesis of upper airway collapse in OSA involves fixed and dynamic factors. Fixed (structural) factors include obesity, craniofacial abnormalities, and anatomical variations in the upper airway. Men typically have longer and more collapsible upper airways, contributing to their higher prevalence of OSA [25,26]. With ageing, increased upper airway closing pressure results from decreased muscle tone, reduced tissue elasticity, diminished peripheral receptor sensitivity, and fat deposition in the upper airway, further increasing susceptibility to OSA [25,26]. Additional structural risk factors include airway stenosis, soft tissue hypertrophy, and alterations in the hyoid bone, maxilla, and mandible [15]. Dynamic (functional) factors include neuromuscular responsiveness, arousal threshold, upper airway and lung volume, loop gain, and critical closing pressure [15,25]. During sleep, the redistribution of fluid from the lower extremities to the cervical region, especially in individuals with heart failure, can elevate peripharyngeal pressure and promote airway collapse [15,25]. Chronic gastroesophageal reflux, common in OSA patients, may induce bronchoconstriction and decreased lung volumes, undermining upper airway stability. Obesity contributes to a reduced functional residual capacity (FRC), particularly in the supine position, leading to increased airway resistance and a risk of obstruction [15,25,27]. A higher FRC exerts greater caudal traction on the upper airway, enhancing its patency. Conversely, a reduced FRC, typical in obesity, facilitates airway collapse [27]. These complex interrelations between anatomy, fluid dynamics, and lung mechanics underscore the multifactorial nature of OSA.

### 2.2. Sleep Fragmentation and Ventilatory Control in Obstructive Sleep Apnoea

Sleep fragmentation, resulting from recurrent arousals or microarousals, prevents progression into the N3 and REM sleep phases, contributing to daytime somnolence, fatigue, and an increased risk of systemic comorbidities [15,24]. Though sleep fragmentation is a hallmark of OSA, it is also observed in other sleep and psychiatric disorders [24].

In OSA, individuals often display abnormalities in their arousal threshold and ventilatory control [15,25]. The arousal threshold is typically reduced, functioning as a protective mechanism against fatal apnoeic events [15,25,28]. However, a low arousal threshold can paradoxically exacerbate airway instability by inducing premature awakenings, interrupting the compensatory actions of upper airway dilator muscles [28]. This is linked to a concept known as high loop gain, a measure of ventilatory control sensitivity [15,25,28,29]. Loop gain consists of three interdependent components: the respiratory system’s sensitivity to changes in CO_2_, the efficiency of ventilation in correcting CO_2_ levels, and the time delay between blood gas fluctuations and the ventilatory response [27]. In patients with high loop gain, even minor elevations in CO_2_ can provoke an exaggerated ventilatory response, causing hypocapnia. This, in turn, diminishes upper airway muscle tone, increasing the risk of airway collapse [15,25,28,29].

During wakefulness, the tonic activity of the pharyngeal dilator muscles, facilitated by noradrenergic input from the locus coeruleus and serotonergic input from the raphe nuclei, helps maintain airway patency [30,31]. However, these cortical drives decline during sleep, predisposing the airway to collapse. CO_2_ levels modulate the upper airway tone through input from mechanoreceptors and chemoreceptors in the genioglossus muscle that project to the brainstem [15,25]. The dysfunction of this regulatory axis results in decreased dilator pressure and increased resistance, reinforcing airway collapse. Increased adiposity and extracellular fluid accumulation in the cervical and thoracic regions further impair this mechanism, exacerbating sleep-related breathing instability [32].

### 2.3. Intermittent Hypoxia, Oxidative Stress, and Systemic Inflammation

The cyclical episodes of intermittent hypoxia, characteristic of OSA, often lasting up to 60 s and recurring nightly over extended periods, are central to its pathophysiology. Unlike chronic lung diseases, where hypoxia is prolonged and stable, intermittent hypoxia involves repetitive desaturation–reoxygenation cycles, which intensify oxidative stress and inflammation [33].

At the molecular level, intermittent hypoxia induces the excessive production of ROS, which triggers widespread oxidative damage [15,16,33]. The transcription factor HIF-1α is important in orchestrating cellular responses to hypoxia. Under normoxia, HIF-1α is rapidly degraded; during hypoxia, it stabilises, translocates to the nucleus, and activates the transcription of genes essential for hypoxic adaptation [16,34]. Intermittent hypoxia also increases HIF-1α transcription via the ROS-mediated activation of calcium-dependent signalling pathways [15,16,34,35]. In contrast, intermittent hypoxia promotes the degradation of HIF-2α, which normally facilitates the production of antioxidant enzymes, rendering tissues more vulnerable to oxidative injury [15,34,35].

Intermittent hypoxia upregulates immediate early genes such as c-fos, enhances sympathetic nervous system activity, and promotes systemic inflammation [15,34,35]. The sensitisation of the carotid body to hypoxia augments the sympathetic output, contributing to sustained hypertension and autonomic dysregulation [15,34,35]. These mechanisms underlie many OSA-associated comorbidities, including cardiovascular disease, arrhythmia, vasospasm, and metabolic dysfunction [33,36]. Intermittent hypoxia also disrupts glucose metabolism via oxidative stress and sympathetic overactivation [15,33,36] and contributes to neurocognitive impairments through hippocampal neuronal injury [37].

OSA is increasingly recognised as a systemic inflammatory disorder. ROS directly damage endothelial and epithelial tissues and activate inflammatory pathways such as NF-κB in neutrophils, leading to the heightened expression of proinflammatory cytokines [38]. Histological studies have revealed oedema and macrophage infiltration in the upper and lower airway mucosa of OSA patients [38]. Furthermore, innate and adaptive immune responses are upregulated [38], contributing to endothelial dysfunction, increased vasoconstrictor tone, and atherosclerosis [38,39].

Recent evidence suggests that upper airway dysbiosis in OSA may amplify systemic inflammation by elevating circulating cytokine levels, creating a vicious cycle of oxidative stress, immune activation, and airway instability [40]. These inflammatory and oxidative processes exacerbate OSA and contribute to its neuro-ophthalmological manifestations, including optic nerve and retinal vascular alterations.

## 3. Neuro-Ophthalmological Manifestations of Obstructive Sleep Apnoea

Growing evidence indicates a significant association between OSA and a spectrum of neuro-ophthalmological disorders, underpinned by shared pathophysiological mechanisms such as intermittent hypoxia, oxidative stress, dysregulated vascular autoregulation, and systemic inflammation. These interconnected processes adversely impact the optic nerve and central visual pathways, contributing to transient and potentially irreversible visual disturbances. Such changes are frequently overlooked, yet they can profoundly impair visual function, diminish quality of life, and negatively influence long-term outcomes. These findings underscore the critical importance of early detection, timely ophthalmological evaluation, and interdisciplinary management in patients with OSA to prevent or mitigate visual morbidity [7,13,41,42,43].

OSA has been linked to several neuro-ophthalmological conditions, including optic neuropathy, especially NAION; papilledema due to increased intracranial pressure; glaucoma, particularly normal-tension glaucoma; visual field defects; and structural and functional changes in the visual pathways. These disorders may present with acute or chronic visual loss, transient obscurations, or subtle deficits in visual processing [7,13,44]. Table 2 presents an overview of neuro-ophthalmological disorders associated with OSA [7,12,13,14,17,41,42,43,44,45,46,47,48,49,50,51,52,53,54,55,56,57,58,59,60,61,62,63,64,65,66,67,68,69,70,71,72,73,74,75,76,77].

Neuro-ophthalmological disorders can significantly impair daily functioning, especially in patients who are unaware of their condition or remain untreated. Sudden vision loss, as in NAION; progressive field loss, as in glaucoma; or fluctuating vision due to papilledema may reduce driving ability, reading skills, and job performance [7,13,42,43,44]. Additionally, coexistent cognitive deficits often seen in OSA can compound visual processing difficulties [74,75]. The estimated prevalence of neuro-ophthalmological disorders among OSA patients varies, with studies reporting up to 70–80% of NAION patients having underlying OSA [78,79,80].

### 3.1. Optic Neuropathies and Papilledema

One of the most clinically significant neuro-ophthalmological complications of OSA is optic neuropathy, particularly NAION, which is the most common acute optic neuropathy in individuals over 50 years of age. NAION is characterised by sudden, painless vision loss due to the infarction of the anterior optic nerve. OSA is a well-established risk factor for NAION [45,46,47,48]. The underlying mechanisms are multifactorial and include nocturnal hypoxia, impaired optic nerve perfusion, and the dysregulation of autoregulatory mechanisms during sleep [43,44]. During apnoeic episodes, fluctuations in blood pressure and hypoxia contribute to transient optic nerve head ischemia. Additionally, decreased nocturnal perfusion pressure and impaired cerebrovascular reactivity can exacerbate the risk of ischemic injury. The optic nerve head, particularly in individuals with a “disc-at-risk” (small and crowded optic disc), is particularly susceptible to these changes [14,48]. Bilateral or sequential NAION is more common in patients with OSA, and untreated OSA may increase the risk of recurrence in the fellow eye [12,41,49,50,51].

OSA has also been associated with papilledema, particularly in patients with IIH. Although the causal relationship between OSA and increased intracranial pressure remains debated, it is hypothesised that hypoventilation and hypercapnia during apnoeic episodes increase cerebral blood flow and intracranial pressure, thereby contributing to optic disc oedema. Some studies suggest an association between OSA and IIH, particularly in obese women [7,52,53].

The recognition of OSA in patients with IIH is essential, as the treatment of OSA may contribute to improved control over intracranial hypertension and visual outcomes. Although not all OSA patients develop papilledema, those with comorbid IIH may benefit from sleep apnoea evaluation and treatment, which can improve intracranial pressure control and preserve visual function [13,54].

### 3.2. Glaucoma

A growing body of literature supports a significant association between OSA and glaucoma, particularly NTG. Several population-based studies and meta-analyses have reported a higher prevalence of OSA in glaucoma patients and vice versa [55,56,57]. As the most prevalent in OSA patients, NTG is characterised by progressive optic nerve damage and visual field loss without elevated intraocular pressure (IOP). It shares pathophysiological features with OSA, including vascular dysregulation, oxidative stress, the mitochondrial dysfunction of retinal ganglion cells, and impaired optic nerve perfusion [41,56,58,59].

Patients with OSA may exhibit structural changes in the optic nerve head and RNFL as assessed by OCT, with thinning patterns that mirror glaucomatous damage [60]. Intermittent hypoxia in OSA leads to mitochondrial dysfunction in retinal ganglion cells and the upregulation of apoptotic pathways, potentially contributing to optic nerve degeneration. Moreover, increased nocturnal IOP, reduced ocular perfusion pressure, and endothelial dysfunction may compound glaucomatous damage [12,61,62]. Notably, continuous positive airway pressure (CPAP) therapy may slow glaucoma progression by improving nocturnal oxygenation and perfusion [42,55,56]. These findings suggest that screening for OSA in patients with progressive NTG may be clinically justified despite adequate IOP control.

### 3.3. Retinal Microvascular Changes

OSA is increasingly recognised as a condition that affects retinal microcirculation, with implications for retinal function and as a biomarker of systemic disease severity. Intermittent hypoxia and oxidative stress impair the autoregulatory mechanisms of the retinal and choroidal vasculature, leading to endothelial dysfunction, increased vascular permeability, and microvascular rarefaction [42,63,64,65]. These effects are particularly evident in the RNFL, which is sensitive to fluctuations in oxygenation and perfusion [66,67].

Retinal vascular imaging using OCTA has revealed reduced vessel density in the superficial and deep capillary plexus of OSA patients, especially in the peripapillary and macular regions. These findings were more pronounced in individuals with severe OSA and correlated with the oxygen desaturation index and AHI. Additionally, choroidal thickness changes have been reported, although the results vary depending on disease severity and treatment status [68,69,70].

Microvascular changes may also contribute to diabetic retinopathy progression in individuals with comorbid diabetes and OSA, as intermittent hypoxia amplifies inflammation, VEGF expression, and oxidative injury [17,71]. Thus, retinal imaging may serve as a diagnostic tool and a surrogate marker of systemic vascular injury in OSA.

### 3.4. Visual Field Defects and Processing Abnormalities

Visual field abnormalities are frequently reported in OSA patients, even in the absence of diagnosed glaucoma or optic neuropathy. Common patterns include peripheral field constriction, arcuate defects, and generalised depression. These defects may result from subclinical damage to the RNFL or post-retinal visual pathways caused by chronic hypoxia or microvascular ischemia. Visual field testing can be a sensitive marker for early neuro-ophthalmological involvement in OSA [43,72,73].

Neuroimaging studies in OSA patients have revealed white matter alterations, reduced optic radiations, and reduced visual cortex integrity. Diffusion tensor imaging (DTI) and functional magnetic resonance imaging (fMRI) have demonstrated microstructural alterations in the brain regions responsible for visual processing. This may explain subtle impairments in contrast sensitivity, visual reaction time, and motion detection. These changes are often reversible or partially improved following effective treatment for OSA [14,74,81].

Patients with moderate to severe OSA frequently report symptoms of blurred vision, transient visual obscurations, and increased light sensitivity, particularly in the morning, which may reflect temporary optic disc ischemia, papilledema, or disrupted retinal metabolism following nocturnal hypoxic stress. Oculomotor disturbances such as diplopia or convergence insufficiency have also been observed, potentially due to hypoxia-induced cranial nerve involvement or impaired cortical integration [14,42,76]. Neurocognitive impairment, often reported in OSA, may further contribute to delayed visual reaction times and impaired visuospatial orientation [13,76]. MRI studies have demonstrated microstructural alterations in the optic radiation, visual cortex, and cerebellar white matter of OSA patients, which may underlie visual processing delays and attention deficits [74,77].

### 3.5. Therapeutic Implications for Visual Function

The recognition of neuro-ophthalmological manifestations of OSA is crucial for timely diagnosis and intervention. CPAP therapy remains the gold-standard treatment and has beneficial effects on several ophthalmological outcomes. CPAP may improve optic nerve perfusion, reduce IOP fluctuations, and mitigate retinal vascular abnormalities [42,60,82]. However, some reports suggest that CPAP, particularly at high pressure, may occasionally contribute to raised intracranial pressure or induce ocular surface dryness, underscoring the need for careful monitoring in susceptible individuals [83].

The early identification of visual changes in OSA patients, including OCT/OCTA screening and formal visual field testing, can facilitate intervention before irreversible damage occurs. Close collaboration between sleep medicine specialists, ophthalmologists, and neurologists is essential for comprehensive care [42]. Understanding associations between OSA and neuro-ophthalmological disorders improves patient outcomes through earlier detection and treatment, highlighting the eye as a window to systemic disease. Ongoing research into the ocular manifestations of OSA may uncover new diagnostic biomarkers and therapeutic targets for this prevalent condition.

### 3.6. Diagnostic Evaluation of Neuro-Ophthalmological Disorders in Obstructive Sleep Apnoea

Patients with OSA presenting with visual disturbances or at risk of ocular complications should undergo a structured neuro-ophthalmological assessment. The initial evaluation should include a comprehensive ophthalmic examination that comprises best-corrected visual acuity (BCVA), intraocular pressure (IOP) measurement, and dilated fundus examination to assess optic disc and retinal morphology [14,15].

Structural imaging using spectral-domain OCT provides a high-resolution quantification of RNFL thickness, macular ganglion cell complex, and optic nerve head parameters. OCTA enables the non-invasive assessment of retinal and optic nerve microvasculature, which is frequently altered in OSA patients. Neuroimaging, particularly the MRI of the brain and orbits, ideally with diffusion tensor imaging or CT, is indicated in unexplained optic neuropathy, suspected intracranial hypertension, and oculomotor deficits [56,60].

When IIH is suspected, lumbar puncture is indicated to measure opening pressure and exclude secondary causes via cerebrospinal fluid analysis [53]. Ancillary tests such as visual evoked potentials (VEPs), contrast sensitivity testing, and colour vision assessment may aid in evaluating post-retinal visual pathway dysfunction. Given the multifactorial nature of visual symptoms in OSA, interdisciplinary collaboration among ophthalmologists, neurologists, and sleep specialists is recommended for comprehensive diagnosis and monitoring [13,14,15,78].

## 4. Pathophysiological Mechanisms Linking Obstructive Sleep Apnoea and Neuro-Ophthalmological Disorders

OSA may affect the visual system through interconnected mechanisms, including intermittent hypoxia, vascular dysregulation, oxidative stress, inflammation, and fluctuations of intracranial pressure. Understanding these mechanisms helps explain OSA-related neuro-ophthalmological disorders and highlights the importance of early detection and prevention. The key mechanisms are summarised in Table 3, [12,13,14,15,16,18,19,20,21,22,32,38,41,42,44,59,62,65,67,84,85,86,87,88,89,90,91,92,93,94,95,96,97,98,99,100].

### 4.1. Hypoxia-Induced Molecular Changes in Ocular Tissues

Due to their high metabolic demands, the retina and optic nerve are particularly vulnerable to hypoxia. In OSA, recurrent episodes of intermittent hypoxia initiate molecular changes in retinal neurons, ganglion cells, and the optic nerve, leading to visual impairments [87]. While these tissues may tolerate short-term hypoxia [88], prolonged exposure activates signalling pathways [16,87,88].

A key response to hypoxia is the upregulation of HIFs, including HIF-1α, HIF-2α, and HIF-3α, which regulate genes involved in cell survival, glucose metabolism, and angiogenesis. HIF-1α, although protective in early stages, can also initiate pro-apoptotic cascades [88,89,90]. HIF-1α and HIF-2α are key regulators of VEGF-A expression [16,90], promoting angiogenesis. Excessive VEGF activity contributes to the pathological neovascularisation seen in conditions such as diabetic retinopathy, including vascular leakage, haemorrhage, and retinal detachment [88,89]. Targeting HIF-1α pathways shows therapeutic potential in mitigating hypoxia-induced neuro-ophthalmic damage [89].

### 4.2. Intracranial Pressure Fluctuations

OSA is associated with transient and sustained elevations in ICP, which contribute to neuro-ophthalmological pathology [13]. ICP may acutely rise to 90 mmHg following apnoeic episodes [13,93], with higher morning values (~20.7 mmHg) that decline by evening (~17.7 mmHg). Increases are more marked during REM sleep and coincide with oxygen desaturation [13,94],

These fluctuations result from hypercapnia-induced vasodilation, elevated central venous pressure due to forced expiration, and comorbid hypertension [95]. In some cases, OSA presents as a secondary form of IIH, reported in 4–60% of IIH patients [93]. Diagnostic criteria include clinical signs of elevated ICP, an absence of neurological deficits (except abducens palsy), normal neuroimaging aside from ICP-related changes, elevated CSF opening pressure, and the exclusion of secondary causes [93]. Persistent ICP elevation may cause papilledema through impaired axonal transport at the optic disc [13,94,95]. CPAP mitigates ICP fluctuations [94,95], potentially preventing vision loss. Thus, OSA should be considered in unexplained optic disc oedema, particularly in obese individuals, where weight loss offers a dual therapeutic benefit [13,94].

### 4.3. Endothelial Dysfunction and Vascular Dysregulation

Intermittent hypoxia in OSA induces oxidative endothelial damage by increasing ROS and inhibiting nitric oxide via endothelial nitric oxide synthase phosphorylation [22,96]. COX-2 upregulation and elevated endothelin-1 (ET-1) contribute to vascular inflammation and vasoconstriction, impairing retinal ganglion cell survival and optic nerve function [22,65,67].

Ocular blood flow autoregulation, which relies on stable perfusion and oxygenation, is disrupted by hypoxia-driven vasodilation and imbalanced vasoactive mediators in OSA [15,16,22,65,96]. HIF-1–mediated VEGF expression promotes fragile neovessels prone to leakage and haemorrhage [16,89].

A rat model exposed to intermittent hypoxia showed enhanced ET-1 vasoconstriction, reduced NO signalling, and impaired endothelial vasodilation, confirming key mechanisms of vascular dysregulation in OSA [91]. Chronic endothelial dysfunction also promotes atherosclerosis, compromising optic nerve perfusion and autoregulation [42].

### 4.4. Chronic Inflammation and Oxidative Stress

Intermittent hypoxia stabilises HIF-1α, increasing ROS production, which leads to mitochondrial damage, apoptosis, and extracellular matrix (ECM) degradation in ocular tissues [42,44,87,88,89,97]. HIF-1α and NF-κB activation enhances inflammation via TNF-α, IL-6, and ICAM-1 [21,92,97].

The proinflammatory environment contributes to OSA-related neuro-ophthalmological conditions [13,42,44,65,67,87,88,89,91]. In NAION, hypoxia contributes to neurovascular compromise and local inflammation via TNF-α and IL-6 [14]. Glaucoma involves microglial activation and cytokine-mediated neuronal damage, while VEGF and ICAM-1 promote the progression of diabetic retinopathy [14]. OSA-induced hypercoagulability may result in retinal vein occlusion [44].

Due to low antioxidant defences, retinal ganglion cells and optic nerves are vulnerable to ROS. Oxidative stress impairs nitric oxide signalling and increases endothelin-1, exacerbating ischemia [18,98].

Hypoxia-induced matrix metalloproteinase (MMP) upregulation disrupts the ECM, contributing to floppy eyelid syndrome and exfoliative glaucoma in OSA [19,59].

### 4.5. Neurodegenerative Pathways and Protective Mechanisms

Glaucoma and optic neuropathy are increasingly recognised as neurodegenerative disorders [100]. Their pathogenesis involves glutamate excitotoxicity, oxidative stress, reduced optic nerve perfusion, microglial activation, and decreased brain-derived neurotrophic factor (BDNF), leading to retinal ganglion cell damage [65,84,85,86,98]. Potential neuroprotective strategies for OSA-related degeneration include enhancing BDNF signalling and antioxidant defences [85,86,99].

## 5. Clinical Management Strategies

OSA is increasingly recognised as a multisystem disorder with profound implications for neuro-ophthalmological health. Given its chronic nature and systemic associations, therapeutic strategies must effectively alleviate immediate symptoms and be capable of reducing long-term complications. Current clinical management options include CPAP therapy as the gold standard, presenting alternative treatments and highlighting the importance of multidisciplinary care and patient education. Managing neuro-ophthalmological complications in OSA requires an integrated therapeutic approach that addresses systemic disease mechanisms and ocular manifestations. The evidence suggests that timely and targeted intervention can slow or reverse vision-threatening changes, especially when implemented early in the disease course. Table 4 outlines the therapeutic interventions available for neuro-ophthalmological disorders in the context of OSA [2,7,14,23,36,43,96,101,102,103,104,105,106,107,108,109,110,111,112,113,114,115,116,117,118].

### 5.1. Continuous Positive Airway Pressure Therapy

**Figure 1:**
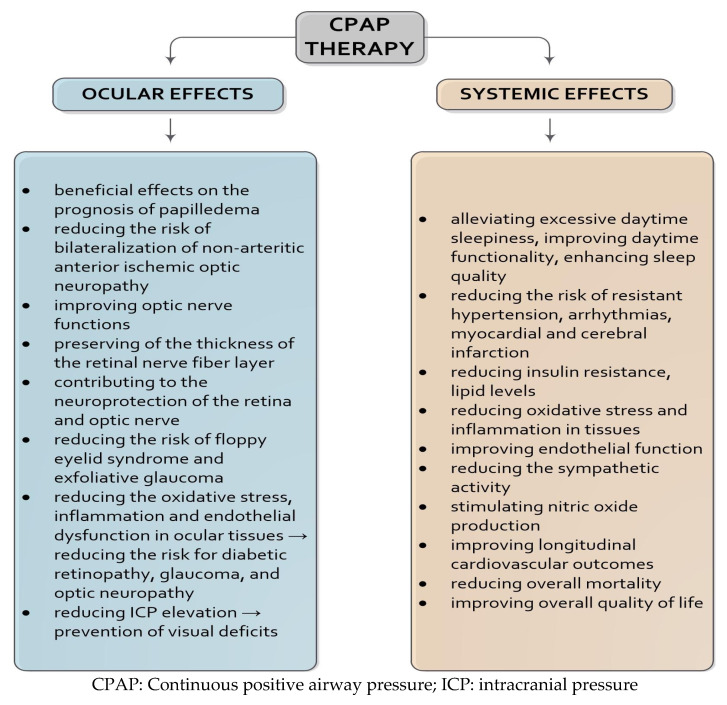
Summary of the ocular and systemic effects of CPAP therapy.

CPAP therapy remains the cornerstone in managing moderate to severe OSA due to its efficacy in improving short- and long-term outcomes [2,36]. Clinically, CPAP effectively reduces excessive daytime sleepiness, enhances daytime function, and improves overall sleep quality, often within just a few days of initiation [2,112]. Beyond symptomatic relief, CPAP therapy has demonstrated systemic benefits, particularly in cardiovascular health. It has been shown to lower the risk of resistant hypertension, arrhythmias, and myocardial and cerebral infarction [2,36,113]. In addition to the systemic, CPAP has also significant ocular effects (Figure 1) [2,23,36,96,103,112,113,114,115,116,117].

Mechanistically, CPAP maintains upper airway patency by delivering a constant positive pressure throughout the respiratory cycle, preventing airway collapse during sleep [2]. This positive pressure extends into the pulmonary alveoli, increasing functional residual capacity, reopening collapsed alveoli, and enhancing gas exchange by improving the ventilation–perfusion ratio [114,115]. This redistribution of blood flow away from poorly ventilated lung regions further supports systemic oxygenation [114,115].

A standard CPAP setup includes a pressure-generating unit and a mask, which can be nasal, oronasal (full-face), or a nasopharyngeal tube [114]. CPAP is contraindicated in patients with pneumothorax, impaired consciousness, or an inability to breathe spontaneously [114]. Despite its benefits, patient adherence remains a major challenge. Common barriers include mask discomfort, skin irritation, nasal dryness, mucosal inflammation, aerophagia, and eye complications such as dryness, conjunctivitis, or irritation caused by air leakage [103,114]. These adherence issues have prompted alternative treatment approaches [103].

### 5.2. Alternative Therapies: Surgical, Pharmacological, and Lifestyle Interventions

Alternative treatments may be considered for patients with mild to moderate OSA or those intolerant to CPAP. These include surgical options that aim to modify and reduce the collapsibility of the upper airway structures [2,103,114]. Uvulopalatopharyngoplasty, the most common surgical approach, involves the removal of the uvula, soft palate, and tonsils to enlarge the oropharyngeal airway [2,104]. However, its long-term success in severe OSA is limited, and it may compromise future CPAP effectiveness by encouraging mouth breathing [2,104]. Side effects include postoperative pain, dysphagia, altered voice, and a foreign body sensation. As a result, newer, less invasive techniques such as lateral or reposition pharyngoplasty and hybrid palatal surgeries are being adopted to minimise complications while preserving efficacy [104]. Maxillofacial surgeries, particularly maxillary advancement via Le Fort I osteotomy, show promise in younger patients with craniofacial abnormalities and lower BMI or AHI scores; however, CPAP remains more effective in severe OSA [2,105]. Mandibular advancement devices (MADs) offer a non-invasive alternative that increases pharyngeal space by protruding the lower jaw and demonstrate good efficacy in reducing daytime symptoms, especially in patients with mild OSA [2,106].

Pharmacologic agents targeting daytime sleepiness or neuromuscular tone in the upper airway are emerging as adjunctive options. Modafinil, armodafinil, pitolisant, and solriamfetol have demonstrated efficacy in enhancing wakefulness by modulating central nervous system neurotransmitters [108,109]. Solriamfetol, a selective dopamine and norepinephrine reuptake inhibitor, is approved in Europe for OSA-related excessive sleepiness, though it requires caution in hypertensive patients [109]. Agents such as reboxetine or atomoxetine combined with oxybutynin may improve upper airway tone [108]. Tirzepatide, a dual GLP-1/GIP receptor agonist recently approved by the FDA, has been endorsed as the first pharmacotherapy for moderate to severe OSA in obese adults, offering dual weight loss benefits and an improved metabolic profile [110]. However, further longitudinal trials are needed to establish the safety and long-term efficacy of these agents [108].

Lifestyle modification, primarily weight reduction, also plays a pivotal role. Although weight loss rarely normalises the AHI, it substantially improves outcomes when combined with CPAP [2,107]. Meta-analyses suggest a 20% BMI reduction leads to an approximately 57% improvement in the AHI [107]. However, even post-bariatric surgery, many patients continue to require CPAP due to residual OSA [2,85,99,102,107,108,118].

### 5.3. Antioxidants and Neuroprotective Agents

Oxidative stress is a key contributor to the pathogenesis of OSA-related neuro-ophthalmological complications, promoting retinal ganglion cell damage, optic nerve dysfunction, and microvascular impairment [14,15,59,63,65,97,102]. Antioxidants, including vitamins C and E, glutathione, and resveratrol, have shown potential in reducing oxidative damage and enhancing cellular resilience in experimental models of intermittent hypoxia. Additionally, neuroprotective agents targeting mitochondrial integrity and inflammatory cascades are being investigated for their potential to preserve visual function in this context. Clinical studies suggest that antioxidant supplementation may improve endothelial function, reduce oxidative burden, and enhance sleep quality in patients with OSA [15,85,86,99,102,108,118]. A recent systematic review and meta-analysis confirmed the beneficial effects of antioxidant therapy on the oxidative biomarkers and sleep parameters in this population [15,102,111]. Further studies are needed to evaluate their long-term safety and efficacy in preventing or slowing optic nerve damage in this population.

### 5.4. Diagnostic and Prognostic Significance of Polysomnography

OSA diagnosis is primarily based on clinical symptom assessment and confirmed through overnight polysomnography, which records a wide range of physiological parameters during sleep. These include oxygen desaturation, sleep architecture, the REM/NREM sleep ratio, body position, and limb movements. The most commonly used index derived from polysomnography is the AHI, which remains the cornerstone for diagnosing and grading the severity of OSA [119]. Nevertheless, reliance on the AHI alone may underestimate the complexity and heterogeneity of OSA. Distinct endo/phenotypes such as those characterised by severe nocturnal hypoxemia or prominent periodic limb movements may have a disproportionately higher risk of cardiovascular and neurovascular complications, independent of AHI severity [119,120]. Advanced polysomnography phenotyping has revealed variability in disease mechanisms and trajectories, providing a foundation for more individualised treatment strategies [120,121].

Polysomnography also holds significant value in identifying potential neuro-ophthalmological complications of OSA. Patients diagnosed with NAION demonstrate a significantly increased prevalence of OSA [13,122]. In light of this association, some authors recommend routine polysomnography screening in all patients diagnosed with NAION to improve early detection and management [13].

### 5.5. Multidisciplinary Care and Patient-Centred Management

Optimal OSA care, particularly in patients with neuro-ophthalmological involvement, necessitates a multidisciplinary, patient-centred approach. Otorhinolaryngologists, pulmonologists, ophthalmologists, sleep specialists, nutritionists, psychologists, and psychiatrists should collaborate to tailor interventions that improve quality of life and minimise disease burden [2,114]. The increasing recognition of OSA as a heterogeneous disorder has led to precision-based treatment models, considering individual endotypes, phenotypes, and gender-specific therapeutic responses [2,120].

Patient education is critical for enhancing adherence and outcomes. Educational initiatives should inform patients about disease mechanisms, therapeutic options, and the consequences of untreated OSA. Studies confirm that patient education significantly improves CPAP compliance [116]. Animal studies using vitamins and antioxidants show reduced oxidative damage, but further clinical validation is required [99]. CPAP therapy reduces inflammatory markers and restores vascular function, indirectly protecting retinal and optic nerve integrity [14,123]. Continued investigation into the neuroprotective effects of CPAP and adjunctive therapies is essential. Combining education with motivational interviewing, peer support, and real-time feedback from modern CPAP devices further strengthens adherence and engagement [117]. Today’s CPAP machines often provide simplified sleep reports and therapy feedback, empowering patients and facilitating ongoing communication with healthcare providers [117].

## 6. Prognostic Impact of CPAP Therapy on Neuro-Ophthalmological Outcomes

### 6.1. Neuroprotective Effects and Clinical Impact of CPAP Therapy

There is robust evidence that the treatment of OSA, particularly through CPAP therapy, can significantly mitigate the severity and frequency of its neuro-ophthalmological symptoms [44]. CPAP therapy reduces systemic oxidative stress and inflammation, key contributors to neurovascular injury. These effects are mediated through reductions in inflammatory and oxidative markers such as CRP, IL-6, TNF-α, and nitric oxide [12,42,44,97]. CPAP also improves endothelial function, lowers systemic blood pressure by reducing sympathetic tone, enhances nitric oxide bioavailability, and improves insulin sensitivity, improving neurological outcomes [44,96,97].

Adjunctive antioxidant supplementation (glutathione, vitamin C, and vitamin E) has been shown to further decrease oxidative stress in patients with OSA undergoing CPAP therapy, potentially enhancing outcomes in comorbid neuro-ophthalmological diseases [97,99]. Importantly, CPAP adherence is linked to a decreased neutrophil-to-lymphocyte ratio, a biomarker of systemic inflammation, suggesting a reduced inflammatory burden in compliant patients [124]. Surgical alternatives to CPAP also reduce inflammatory markers, although to a lesser extent [96,97].

The benefits of CPAP therapy extend to various neuro-ophthalmological conditions, with case reports and observational studies demonstrating improvements in IIH and the resolution of papilledema across treatment modalities [95]. Although some controversy remains regarding the potential of CPAP to elevate intraocular pressure in glaucoma patients, the current evidence does not contraindicate its use in this population [14,114]. Longitudinal studies support the protective effect of CPAP in NAION. A three-year follow-up study found that patients with severe OSA who were non-adherent to CPAP therapy had a significantly higher risk of contralateral NAION compared to both moderate OSA patients and controls without OSA [65]. In contrast, CPAP-adherent patients demonstrated no increased risk compared to the controls [65].

Electrophysiological studies further confirm CPAP’s neuroprotective effects. A study reported the normalisation of visual evoked potentials (VEPs), including improved P100 amplitudes and reduced latencies, in patients who adhered to CPAP therapy for one year, with no improvements observed in patients who did not adhere to the treatment [125]. Another study reported a significantly thinner nasal RNFL in their non-CPAP group, further supporting the role of CPAP in preserving optic nerve integrity [58].

### 6.2. Adherence to CPAP and Disease Progression

Despite its benefits, long-term CPAP adherence remains a challenge. An eight-year follow-up study reported no significant differences in mortality or hospitalisation between adherent and non-adherent patients [126]. However, recent meta-analyses indicate that consistent CPAP use, particularly over four hours per night, may reduce all-cause mortality [2,36,113,126]. This underscores the need for high-quality longitudinal studies on neuro-ophthalmological outcomes [2,14]. Therapeutic success in OSA is closely tied to adherence, commonly defined as CPAP use ≥4 h per night on ≥70% of nights over 30 days [126]. In practise, adherence varies widely, with 29–83% of patients falling below this threshold [127].

Several factors influence adherence. A large multinational study involving 275 OSA patients with cardiovascular comorbidities identified early CPAP usage and the number of side effects at one month as the strongest predictors of long-term adherence [128]. Patients with more pronounced daytime sleepiness or frequent sleep-related hospitalisations were also less likely to adhere to therapy [126]. Moreover, adherence patterns stabilise early, often within the first week of treatment, highlighting the need for immediate interventions to improve compliance [128]. Nonadherence is particularly detrimental in patients with severe OSA and NAION, where it significantly increases the risk of contralateral eye involvement [44,58]. Despite this, the literature on adherence and its direct impact on neuro-ophthalmological outcomes remains scarce [14,58,116].

### 6.3. Monitoring Strategies and the Role of OCT

Patients with neuro-ophthalmological diseases should undergo regular polysomnography [13], but likewise, patients with OSA should have regular ophthalmological examinations [14,60]. Routine monitoring using polysomnography and ophthalmic imaging is crucial. Patients with OSA should undergo regular ophthalmologic examinations, including OCT, to monitor disease progression and treatment response [14,60]. A prospective study assessing macular thickness, RNFL, and the optic nerve head before and after OSA treatment found that CPAP and surgical interventions improved retinal architecture. Specifically, treatment reduced retinal swelling in mild to moderate OSA and reversed atrophy in severe cases. No significant differences were found between treatment modalities. These findings underscore OCT’s utility as a non-invasive biomarker for monitoring OSA-associated neuro-ophthalmological changes and treatment efficacy. Early retinal changes may reveal undiagnosed OSA, positioning OCT as a valuable diagnostic tool [60].

## 7. Future Directions and Research Opportunities

**Figure 2:**
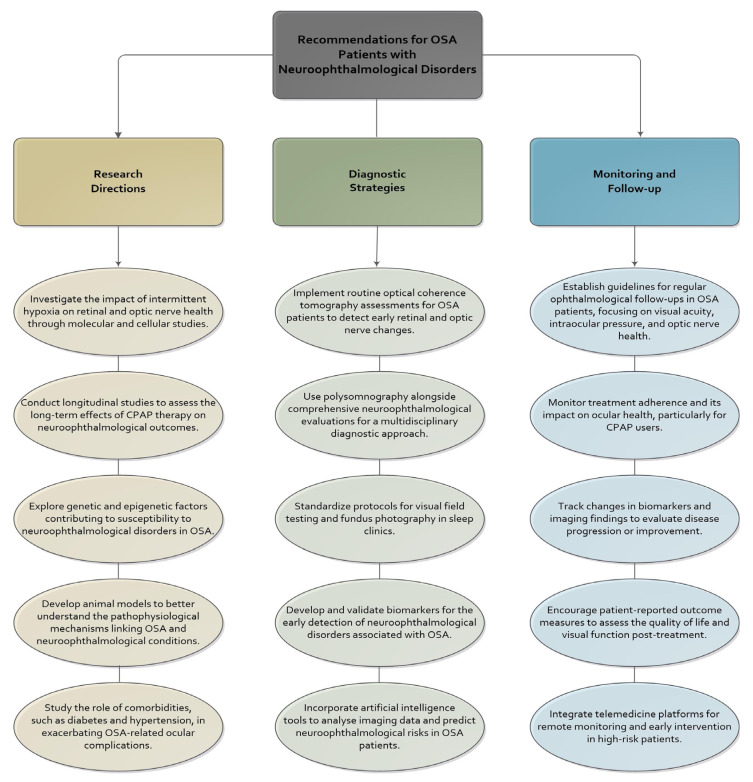
Recommendations for research, diagnosis, and monitoring of neuro-ophthalmological disorders in obstructive sleep apnoea patients.

Despite substantial progress in elucidating the relationship between OSA and neuro-ophthalmological disorders, significant knowledge gaps hinder the development of standardised clinical pathways and targeted therapies. Future research must bridge these gaps by refining our understanding of disease mechanisms, enhancing diagnostic precision, and developing individualised treatment strategies for the heterogeneous nature of OSA and its neuro-ophthalmological manifestations (Figure 2) [13,129,130,131].

A primary research priority is the design and implementation of prospective longitudinal studies that evaluate the long-term effects of OSA therapies, particularly CPAP, on neuro-ophthalmological outcomes. While current evidence suggests CPAP may reduce RNFL thinning, improve VEPs, and slow the progression of conditions such as NAION, most existing studies are limited by short follow-up periods, small sample sizes, and heterogeneity in their diagnostic methods. There is a clear need for large-scale, multicentre trials with standardised ophthalmologic endpoints to assess visual function, structural integrity, and patient-reported visual quality of life [79,82,132].

A related area of opportunity is the integration of ocular biomarkers into OSA screening and monitoring protocols. Advanced imaging modalities such as OCT and OCTA can reveal subclinical changes in optic nerve head perfusion, retinal ganglion cell loss, and microvascular alterations before clinical symptoms arise. These tools should be investigated as diagnostic adjuncts and potential biomarkers for systemic disease severity and treatment responsiveness. In parallel, the application of artificial intelligence (AI) and machine learning in ophthalmology and sleep medicine holds promise for improving the early detection and prognosis of neuro-ophthalmological disorders associated with OSA. The AI-driven analysis of OCT and OCTA images may enable automated screening for RNFL thinning or microvascular changes before clinical symptoms appear. Moreover, the AI-based integration of multimodal data, including sleep parameters, ocular imaging, and systemic biomarkers, could support precision medicine by identifying patient-specific risk profiles and guiding individualised treatment strategies [7,60,132,133].

Understanding the molecular mechanisms linking OSA to neuro-ophthalmological damage is essential for developing targeted therapies. Intermittent hypoxia initiates a cascade of oxidative stress, endothelial dysfunction, and inflammation, implicating factors such as HIF-1α, VEGF, ET-1, MMPs, and neuroinflammatory cytokines. Experimental models, especially those using tissue-specific gene knockout or pharmacologic inhibition, can help clarify the role of these molecules in promoting optic nerve and retinal injury. This knowledge could facilitate the development of adjunctive treatments, including anti-VEGF agents, neuroprotective antioxidants, and inhibitors of inflammatory signalling pathways [33,59,126].

Simultaneously, precision medicine approaches should be explored to stratify OSA patients according to their neuro-ophthalmological risk profiles. Current OSA classification relies heavily on the AHI, which does not fully capture disease complexity. Phenotyping based on endotypes such as high loop gain, low arousal threshold, impaired upper airway tone, and associated biomarkers may allow clinicians to predict which patients are most susceptible to ocular complications. Incorporating machine learning algorithms trained on multimodal data, including polysomnography metrics, genetic markers, imaging features, and symptom profiles, could enhance risk prediction and tailor treatment decisions to individual needs [134,135]. There is also a compelling need to investigate the impact of sex, age, and comorbidities on the ocular manifestations of OSA. While the condition is more prevalent in men, recent data suggest that women may be underdiagnosed due to atypical symptom presentation. Furthermore, ageing and coexisting diseases such as diabetes, hypertension, and obesity may modulate both the risk and severity of visual complications [135,136,137].

Recent developments in OSA pharmacotherapy have introduced new opportunities for ocular outcome research. Agents such as solriamfetol, GLP-1 receptor agonists, and reboxetine oxybutynin combinations have demonstrated systemic benefits, including weight reduction and neuromuscular tone modulation. However, their impact on ocular perfusion, inflammation, and retinal architecture remains unknown. Furthermore, the role of antioxidant supplementation (vitamins C and E, glutathione, resveratrol, or BDNF enhancers) in attenuating intermittent hypoxia-induced oxidative damage should be explored in randomised controlled trials [15,108,118].

There is also a pressing need to establish clinical guidelines that address ocular screening and referral in patients with OSA. The current guidelines focus primarily on cardiovascular, metabolic, and respiratory endpoints, overlooking vision-related consequences. Interdisciplinary consensus statements should advocate routine ophthalmological evaluation, particularly for patients with severe OSA, comorbid diabetes, or visual complaints, and outline criteria for imaging, follow-up intervals, and collaborative care models [101].

Finally, developing interdisciplinary care pathways involving sleep specialists, ophthalmologists, neurologists, and primary care providers will be vital in translating research into practise. Future implementation studies should assess whether integrated management models improve early detection, treatment adherence, visual outcomes, and quality of life. Advancing the field of OSA-associated neuro-ophthalmological disease requires a concerted effort to refine pathophysiological insights, enhance diagnostic and prognostic precision, and expand therapeutic options. Interdisciplinary collaboration, technological innovation, and personalised medicine approaches will be key to addressing the current limitations and improving the quality of life for patients affected by this multifaceted condition.

### Recommendations for Clinical Practice and Future Research

Given the growing evidence base and the systemic complexity of OSA, integrating structured clinical protocols and targeted research initiatives could advance the diagnosis and management of its neuro-ophthalmological manifestations. Clinicians should consider implementing routine ophthalmologic screening, using OCT, OCTA, and standard automated perimetry, in patients with moderate to severe OSA, especially those exhibiting visual symptoms, progressive optic neuropathy, or systemic comorbidities such as diabetes or obesity. The early identification of subclinical retinal and optic nerve changes through these modalities can enable timely intervention and potentially prevent irreversible visual impairment [13,14,15,102]. The therapeutic role of CPAP in stabilising ocular perfusion, reducing oxidative stress, and preserving retinal ganglion cell integrity is well established and should be prioritised in affected patients. Careful monitoring for potential side effects, such as ocular surface dryness or transient increases in intracranial pressure, is warranted [112,114].

The complexity of visual disorders linked to OSA necessitates close collaboration among ophthalmologists, sleep specialists, and neurologists to ensure accurate diagnosis, ongoing monitoring, and the optimisation of individualised treatment plans. Future research should prioritise longitudinal, multicentre studies assessing the effects of OSA treatment modalities such as CPAP, mandibular advancement devices, and pharmacotherapy on neuro-ophthalmologic outcomes using standardised structural and functional metrics, including RNFL thickness, vessel density, and visual evoked potentials [124,125]. Artificial intelligence and machine learning tools offer promising opportunities for the automated detection of subtle ocular changes and risk stratification in high-throughput settings, and their incorporation into diagnostic workflows should be systematically evaluated [7,60,132,133]. In parallel, molecular and translational studies are needed to elucidate the precise mechanisms by which intermittent hypoxia, oxidative stress, endothelial dysfunction, and inflammatory cascades contribute to optic nerve and retinal injury, with the ultimate goal of identifying novel therapeutic targets for neuroprotection in OSA [15,102,108,118]. These efforts will be crucial for bridging existing knowledge gaps and establishing comprehensive, precision-based frameworks to prevent vision loss in this high-risk population.

## 8. Conclusions

OSA is a multifactorial disorder with far-reaching systemic consequences, including a growing spectrum of neuro-ophthalmological complications. As evidence accumulates linking intermittent hypoxia, oxidative stress, inflammation, and vascular dysregulation to optic nerve and retinal damage, integrating ophthalmological assessments into OSA management becomes increasingly justified. Advancements in imaging technologies, molecular profiling, and CPAP therapy offer promising avenues for early detection, personalised risk assessment, and vision preservation. However, major gaps remain in our understanding of long-term outcomes and optimal treatment strategies. Future research should prioritise prospective studies, biomarker discovery, and interdisciplinary care models to improve patient outcomes. Ultimately, addressing the visual consequences of OSA will require a paradigm shift in clinical practise that bridges the respiratory and ocular disciplines to ensure comprehensive and preventive care.

## Figures and Tables

**Table 1:** Classification of OSA severity based on apnoea–hypopnoea index [4].

OSA Severity	AHI (Events/Hour)	Clinical Interpretation
Mild	5–15	May present with mild daytime sleepiness
Moderate	15–30	Increased risk for systemic and ocular complications
Severe	>30	High risk for cardiovascular, neurological, and ocular damage

OSA: Obstructive sleep apnoea; AHI: apnoea–hypopnoea index.

**Table 2:** Neuro-ophthalmological disorders associated with obstructive sleep apnoea.

Ophthalmological Disorder	Pathophysiological Mechanisms	Main Clinical Features	Type of Association with OSA	References
Non-arteritic anterior ischaemic optic neuropathy	Nocturnal hypoxia, impaired optic nerve perfusion, autoregulatory dysfunction	Sudden, painless vision loss; altitudinal visual field defects	Strong epidemiological and biological association	[12,41,42,43,44,45,46,47,48,49,50,51]
Papilledema (especially in IIH)	Increased intracranial pressure during REM sleep, hypercapnia, venous outflow resistance	Transient visual obscurations, headache, bilateral disc swelling	Suggested association, particularly in obese women with IIH	[7,13,52,53,54]
Glaucoma (especially normal-tension glaucoma)	Oxidative stress, endothelial dysfunction, impaired ocular perfusion, mitochondrial damage	Progressive visual field loss, optic nerve cupping; NTG with normal IOP	Strong clinical and pathophysiological association	[7,12,41,42,55,56,57,58,59,60,61,62]
Retinal microvascular changes	IH-induced vascular dysregulation, VEGF overexpression, endothelial dysfunction	Reduced vessel density (using OCTA), RNFL thinning, choroidal thickening	Growing evidence, particularly in severe OSA	[17,42,63,64,65,66,67,68,69,70]
Diabetic retinopathy (in OSA patients with DM)	Enhanced VEGF and inflammation, oxidative stress, disrupted autoregulation	Haemorrhages, neovascularisation, macular oedema	Exacerbation of disease severity and progression	[7,12,14,17,71,72]
Visual field defects	Subclinical RNFL damage, cortical hypoxia	Arcuate or peripheral defects, generalised depression	Subclinical or early-stage manifestation in OSA patients	[12,14,42,43,72,73,74]
Oculomotor dysfunction and visual processing deficits	Hypoxic injury to cranial nerves and visual cortex	Diplopia, convergence insufficiency, delayed visual reaction, impaired contrast sensitivity	Frequently under-recognised, linked to cognitive dysfunction	[12,13,42,74,75,76,77]

IIH: Intracranial hypertension; NTG: normal-tension glaucoma; IOP: intraocular pressure; OSA: obstructive sleep apnoea; IH: intermittent hypoxia; VEGF: vascular endothelial growth factor; OCTA: optical coherence tomography angiography; RNFL: retinal nerve fibre layer; DM: diabetes mellitus.

**Table 3:** Pathophysiological mechanisms linking OSA to neuro-ophthalmological diseases.

Mechanism of Association	Key Molecular Mediators	Mode of Action and Effect	Ocular Consequences	References
Intermittent hypoxia	HIF-1α, HIF-2α, VEGF, ROS	Induces oxidative stress, mitochondrial dysfunction, angiogenesis, and apoptotic pathways	Retinal ganglion cell loss, neovascularisation, oedema	[12,13,14,16,87,88,89,90,91,92]
Intracranial pressure fluctuations	CO_2_, cerebral vasodilation mediators, venous pressure	Elevates ICP, disrupts axoplasmic flow, impairs optic nerve perfusion	Papilledema, optic disc oedema, visual obscurations	[12,13,14,93,94,95]
Vascular dysregulation	ET-1, nitric oxide, eNOS, COX-2	Causes endothelial dysfunction, vasoconstriction, and impaired ocular blood flow	Glaucoma progression, optic nerve ischemia	[15,16,22,41,65,67,89,96]
Systemic and local inflammation	TNF-α, IL-6, IL-8, CRP, NF-κB, ICAM-1, VCAM-1	Promotes leukocyte adhesion, increases vascular permeability, disrupts the blood–retinal barrier	Retinal inflammation and microangiopathy, optic nerve inflammation, gliosis	[13,14,21,42,44,65,67,87,88,89,91,92,97]
Oxidative stress	ROS, mitochondrial damage, depleted antioxidants	Triggers cellular apoptosis, reduces antioxidant defences, impairs neurovascular homeostasis	Retinal ganglion cell apoptosis, RNFL thinning, glaucomatous optic neuropathy	[18,19,20,38,59,62,91,97,98,99]
Neurodegeneration	Microglial activation, glutamate excitotoxicity, BDNF depletion	Leads to axonal injury, synaptic loss, and impaired neurotrophic support	Visual processing deficits, optic nerve degeneration	[12,13,14,84,85,86,98,99,100]
Extracellular matrix remodelling	MMPs, collagen turnover enzymes	Alters ECM integrity, weakens connective tissues	Floppy eyelid syndrome, predisposition to exfoliative glaucoma	[14,19,32,42]

HIF-1α: Hypoxia-inducible factor 1α; HIF-2α: hypoxia-inducible factor 2α; VEGF: vascular endothelial growth factor; ROS: reactive oxygen species; ICP: intracranial pressure; ET-1: endothelin-1; TNF-α: tumour necrosis factor-α; IL-6: interleukin-6; IL-8: interleukin-8; CRP: C-reactive protein; ICAM-1: intercellular adhesion molecule 1; VCAM-1: vascular cell adhesion protein 1; RNFL: retinal nerve fibre layer; BDNF: brain-derived neurotrophic factor; MMPs: matrix metalloproteinases; ECM: extracellular matrix.

**Table 4:** Therapeutic approaches for neuro-ophthalmological disorders associated with obstructive sleep apnoea.

Therapeutic Options	Targeted Mechanisms	Neuro-Ophthalmological Benefits	Limitations	References
Continuous positive airway pressure	Maintains upper airway patency, reduces IH and ICP, improves optic nerve perfusion	Improves retinal and optic nerve oxygenation, slows glaucoma progression, stabilises ocular vasculature	Poor adherence in some patients may cause ocular dryness or an increase in ICP at high pressure levels	[2,23,36,96,103,112,113,114,115,116,117]
Surgical interventions (UPPP, maxillomandibular advancement)	Improve airway patency and reduce OSA severity	Alternative for patients non-compliant with CPAP; may significantly reduce AHI	Invasive procedures with surgical risk; variable long-term efficacy	[2,23,103,104,105,106,114]
Pharmacological agents (solriamfetol, modafinil)	Promote wakefulness via dopamine and norepinephrine reuptake inhibition; may indirectly reduce oxidative stress	Enhance alertness and quality of life in CPAP-intolerant patients	Do not directly treat neuro-ophthalmological complications; risk of cardiovascular side effects	[2,23,108,109,110]
Weight loss and lifestyle modification	Decreases upper airway resistance, reduces systemic inflammation, and oxidative stress	Lowers AHI and systemic disease burden; improves visual and general health outcomes	Requires sustained patient motivation; effects may vary based on comorbidities	[2,23,107]
Antioxidants and neuroprotective agents	Reduce oxidative stress, preserve mitochondrial integrity, reduce inflammation, support ganglion cell survival	Potential to protect retinal ganglion cells and optic nerve from hypoxic injury, may preserve visual function	Limited clinical trial evidence; effects may be modest	[102,108,111,118]
Interdisciplinary care (sleep medicine, ophthalmology, neurology)	Provides integrated management and monitoring	Optimises visual and neurological outcomes	Requires structured care coordination; not always feasible in all settings	[7,14,43,101,102]

CPAP: Continuous positive airway pressure; IH: intermittent hypoxia; ICP: intracranial pressure; ROS: reactive oxygen species; UPPP: uvulopalatopharyngoplasty; AHI: apnoea–hypopnoea index; OCT: optical coherent tomography; VF: visual field; OCTA: OCT angiography.
